# Spray‐Dried Engineered *Escherichia coli* Whole‐Cell Biocatalysts Enable Cell Recycling and In Vivo α‐Ketoglutarate and NADPH Regeneration for Efficient Codeine Manufacture

**DOI:** 10.1002/bit.70270

**Published:** 2026-06-11

**Authors:** Ali Jahanian, Carlos Horacio Luna‐Flores, Xu Li, Sally L. Gras, Robert E. Speight

**Affiliations:** ^1^ School of Mechanical, Medical and Process Engineering, Faculty of Engineering Queensland University of Technology Brisbane Queensland Australia; ^2^ Centre for Agriculture and the Bioeconomy Queensland University of Technology Brisbane Queensland Australia; ^3^ ARC Centre of Excellence in Synthetic Biology Queensland University of Technology (QUT) Brisbane Queensland Australia; ^4^ Faculty of Science Queensland University of Technology (QUT) Brisbane Queensland Australia; ^5^ Department of Chemical Engineering The University of Melbourne Parkville Victoria Australia; ^6^ The Bio21 Molecular Science and Biotechnology Institute The University of Melbourne Parkville Victoria Australia; ^7^ Advanced Engineering Biology Future Science Platform, Commonwealth Scientific and Industrial Research Organization (CSIRO) Dutton Park Queensland Australia

**Keywords:** alkaloid, biocatalyst, biotransformation, cofactor, spray‐dried, whole‐cell

## Abstract

A recombinant whole‐cell biocatalytic approach provides several advantages over isolated enzymes, such as improved enzyme stability, in vivo cofactor regeneration, and the facilitation of cascade reactions within a single cell. Whole‐cell biocatalysis has gained attention in medicinal opioid production, due to its technical and economic advantages. In this study, recombinant *Escherichia coli* producing thebaine 6‐*O*‐demethylase (T6ODM), neopinone isomerase (NISO), and codeinone reductase (COR) were used to convert thebaine into codeine. Spray‐drying was developed to optimize enzymatic activity in dried biocatalytic cells, achieving over 85% enzyme activity retention. These formulated cells were evaluated over multiple recycled reaction cycles that relied on in vivo cofactor regeneration. The native enzyme isocitrate dehydrogenase (IDH) regenerated both α‐ketoglutarate and NADPH, critical for T6ODM and COR activity, respectively. The biocatalysts demonstrated high durability across 10 reaction cycles, with IDH and COR maintaining consistent activity. However, T6ODM exhibited a 40% reduction in activity, aligning with its lower thermal stability. The two‐step bioconversion process yielded an average 68% molar yield of codeine after 10 cycles, with full thebaine consumption, demonstrating efficient cofactor and co‐substrate recycling without further cellular engineering. This study highlights the potential of spray‐dried *E. coli* cells as a sustainable and scalable approach for codeine biomanufacturing from thebaine. These results provide a strong foundation for further industrial application, offering both economic and technical benefits for large‐scale opioid production.

## 1 Introduction

Microbial whole‐cell biocatalysis provides unique advantages for the efficient and low‐cost biosynthesis of value‐added products, such as pharmaceuticals. A variety of genes for heterologous enzymes have been introduced into microbial cell hosts to optimize the translation of proteins of interest, such biocatalysts and other aspects of cellular metabolism, including cofactor balance (Lin and Tao 2017). The findings of various studies demonstrate that metabolically active resting cells may provide higher yields than growing cells in cofactor‐dependent redox biotransformations (Bühler et al. 2008; Julsing et al. 2012; Willrodt et al. 2016). Unlike growing cells, which require carbon and energy for cell proliferation, the residual metabolism of resting cells can be directed toward redox biocatalysis to achieve higher enzymatic activity when supplemented with energy sources. (de Carvalho 2017; Julsing et al. 2012). Since resting cells are washed to remove undesired metabolites from the medium, improved downstream processing and product recovery are expected. Harvested cells can maintain high activity and cofactor regeneration over extended periods and multiple recycling cycles in biotransformation processes (de Carvalho 2017).

**Figure 1:**
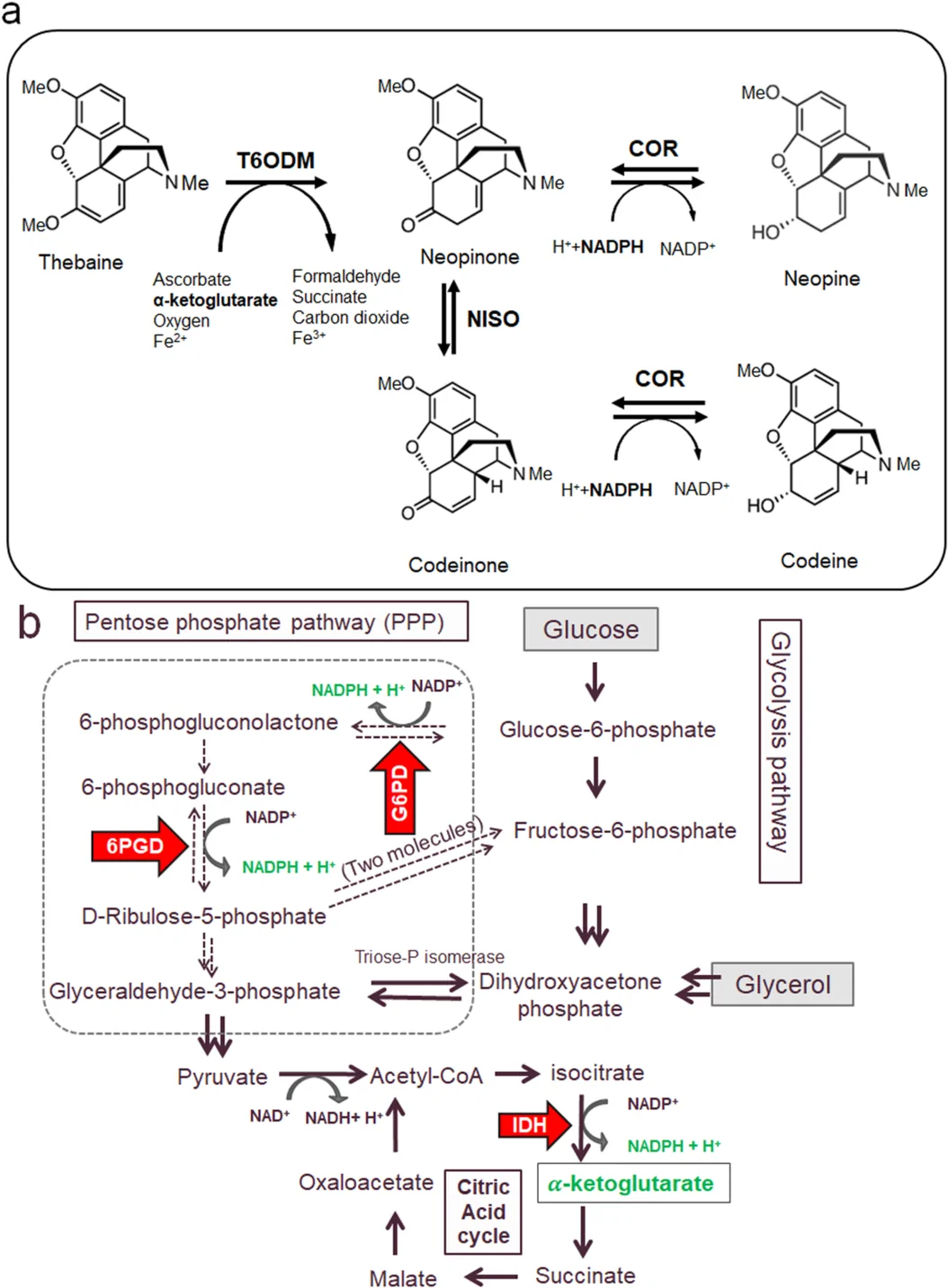
Enzymatic pathway for codeine synthesis from thebaine and for the required cofactors. (a) A three‐step biocatalytic pathway to produce codeine from the precursor thebaine (adapted with permission from Jahanian et al. 2024), and (b) simplified central carbon metabolism through glycolysis, pentose phosphate, and citric acid cycle pathways in *E. coli* cells focusing on NADPH‐generating reactions. Red arrows indicate enzymes that reduce NADP^+^ to NADPH with glucose‐6‐phosphate dehydrogenase (G6PD) and 6‐phosphogluconate dehydrogenase (6PGD) in the pentose phosphate pathway. The third enzyme, isocitrate dehydrogenase (IDH), is part of the citric acid cycle and is located in *E. coli* cytoplasm where it also produces α‐ketoglutarate (2‐oxoglutarate) that is required for T6ODM activity.

Benzylisoquinoline alkaloids (BIAs) are a group of specialized plant compounds known for their diverse chemical structures and biological activities. Among them, codeine and morphine are closely related BIAs widely recognized for their pain‐relieving, or analgesic, effects (Carr et al. 2021). Engineered microbial platforms like *Saccharomyces cerevisiae* and *Escherichia coli* (*E. coli*) have been developed to produce opiates, such as codeine (Dastmalchi et al. 2018; Li et al. 2020a), oripavine (Spencer et al. 2023), and morphine (Galanie et al. 2015) from affordable precursors, such as thebaine using biocatalysis. This commercially viable approach offers a cost‐effective, scalable solution for opiate manufacturing in the pharmaceutical industry. Codeine, an important medicinal opiate, can be synthesized from thebaine through a whole‐cell biocatalytic route using *E. coli* cells (Jahanian et al. 2024; Li et al. 2020b). Figure 1a shows that thebaine is converted to neopinone by the dioxygenase thebaine 6‐*O*‐demethylase (T6ODM), followed by structural isomerization of neopinone to codeinone, which was originally assumed to be spontaneous but is now known to be catalyzed by neopinone isomerase (NISO). Codeinone reductase (COR) is part of the aldo–keto reductase (AKR) superfamily, which is characterized by a triosephosphate isomerase (TIM)‐barrel protein structure. COR reduces codeinone to codeine, or alternatively neopinone to the undesirable product neopine, through reversible NADPH‐dependent. The co‐substrate α‐ketoglutarate for T6ODM and the cofactor NADPH for COR are required for completion of the bioconversion reaction and must be available in stoichiometric quantities for the first and subsequent biocatalyst recycling cycles. Glucose and glycerol metabolism in *E. coli* produces NADPH through three pathways: two from oxidative steps of the pentose phosphate pathway (PPP) and one from the citric acid cycle via isocitrate dehydrogenase (IDH) (Figure 1b). IDH catalyzes the conversion of isocitrate to α‐ketoglutarate, providing the required co‐substrate for the biocatalytic activity of T6ODM (Walton and Stewart 2004), as well as NADPH required for COR activity.

An example of biocatalytic conversion using non‐growing *E. coli* involved phenylacetone monooxygenase, an enzyme belonging to the NADPH‐dependent oxidoreductase family, to produce benzyl acetate. Among different carbohydrates used as sources of reducing power for redox cofactors, the yield of benzyl acetate was higher when glycerol was used as the electron donor, likely due to improved regeneration of the NADPH cofactor (van Bloois et al. 2012).

Another study used a monooxygenase in non‐growing *E. coli* cells to produce lactones, reporting 20 times higher volumetric productivity and shorter reaction completion time for cells harvested in the stationary phase compared with cells harvested in the log phase (Clouthier and Kayser 2007). Another study also reported that cellular NADPH regeneration could sustain stoichiometric cofactor supply for cyclohexanone monooxygenase, resulting in a final caprolactone yield 20 times higher in non‐growing cells than in growing cells (Walton and Stewart 2004). This result was explained by substrate mass transfer through the membrane being rate‐limiting. The membrane permeability of non‐growing bacterial cells was suggested to be greater than that of growing cells, facilitating more efficient mass transfer, which is critical for reducing NAD(P)^+^ cofactors through *E. coli* intracellular metabolic pathways. In the same study, IDH was identified as the major producer of NADPH, and the redox balance was retained throughout the reaction (Walton and Stewart 2004).

The commercial use of enzyme biocatalysts requires formulation in a way that maintains activity during transportation and storage (Cavalcanti et al. 2020). Spray drying is one of the most efficient techniques for long‐term preservation and shipment of microbes at an industrial scale, as it can be operated continuously and at low process cost. This technique is based on the evaporation of water during the atomization of a homogeneous cellular slurry inside a drying chamber (Lee et al. 2018; Salleh et al. 2014), with spray‐dried resting cells shown to deliver higher survival rates than cells harvested while still growing (Pispan et al. 2013; Radulović et al. 2017). Significantly, there is no evidence on the effect of spray drying on recycled resting cells used for biotransformation, or on the availability of nicotinamide redox cofactors and intracellular metabolites in reconstituted spray‐dried *E. coli*. Moreover, the thermal and storage stability of in vivo produced T6ODM and COR enzymes could be a major issue for the sustainability of commercial‐scale bioprocess and has not been studied to date. Therefore, this study aims to investigate redox cofactor availability during whole‐cell recycling, as well as the thermal sensitivity and long‐term preservation of T6ODM and COR enzymes in spray‐dried cells.

In our previous work, we optimized the whole‐cell biocatalyst production using fed‐batch fermentation, achieving an 80% molar yield of codeine from thebaine in a one‐step biotransformation process (Jahanian et al. 2024). This study develops a spray‐dried *E. coli* whole‐cell biocatalyst formulation for reusable codeine biomanufacturing. The main focus was to preserve the activity of T6ODM, NISO, and COR during spray drying, storage, and repeated biotransformation cycles. The study also examined whether the rehydrated resting cells could maintain the intracellular metabolism needed to regenerate α‐ketoglutarate and NADPH in vivo. By combining formulation optimization, enzyme stability analysis, intracellular metabolite analysis, and recycled whole‐cell bioconversion, this work evaluates the potential of spray‐dried *E. coli* cells as a stable and reusable platform for converting thebaine to codeine without external cofactor or co‐substrate regeneration.

## 2 Materials and Methods

Thebaine, codeinone, and codeine were provided by Sun Pharmaceutical Industries Australia Pty Ltd. Analytical grade glucose, glycerol, lactose, sodium ascorbate, α‐ketoglutarate, isopropyl β‐D‐1‐thiogalactopyranoside (IPTG), and protein extraction reagent (BugBuster) were purchased from Merck. Other chemicals and reagents were purchased from Sigma‐Aldrich unless otherwise noted.

### 2.1 Plasmids, Host Bacterial Strain, and Transformation

*E. coli* BL21 (DE3) (Novagen, Germany) harboring the individual plasmids pET‐duet‐1‐PsT6ODM‐PsNISO, and pET28a‐PbCORoptE1‐1 to translate T6ODM/NISO, and COR, respectively, were provided by Sun Pharmaceutical Industries Australia Pty Ltd and the University of Melbourne. Both plasmids were transformed into *E. coli* DH5‐α competent cells for the purpose of cloning and DNA sequencing prior to the transformation of *E. coli* BL21(DE3) competent cells for enzyme production and biotransformation.

### 2.2 Growth Medium Preparation and Cell Cultivation

Defined minimal medium (DMM) was prepared using citrate phosphate buffer composed of citric acid 1.7 g.L^−1^, (NH_4_)_2_HPO_4_ 4 g.L^−1^, KH_2_PO_4_ 13.3 g.L^−1^, which was autoclaved separately at 121°C for 20 min and cooled to room temperature. MgSO_4_ stock solution was then added to final concentration of 2.4 g.L^−1^, together with 1000X thiamine stock solution (0.84 g.L^−1^), and 100X trace element solution containing CoCl_2_.6H_2_O 0.25 g.L^−1^, MnCl_2_.4H_2_O 1.5 g.L^−1^, CuCl_2_.2H_2_O 0.15 g.L^−1^, H_3_BO_3_ 0.3 g.L^−1^, Na_2_MoO_4_.2H_2_O 0.25 g.L^−1^, Zn(CH_3_COO)_2_.2H_2_O 1.3 g.L^−1^, FeSO_4_.7H_2_O 3 g.L^−1^, and EDTA 0.84 g.L^−1^ in deionized water. These solutions were added aseptically after sterilization through 0.2 µm membrane filter. The carbon source solution, such as glucose, glycerol, or lactose was prepared separately, sterilized through a 0.2 µm, and transferred to the conical flasks or bioreactors. Batch cultivation of *E. coli* for 24 h was performed under optimized auto‐induction conditions at 22°C, and 0.9 g.L^−1^ lactose concentrations to produce the required enzymes. The initial carbon source for all fermentations was 20 g.L^−1^.

### 2.3 Thebaine 6‐*O*‐Demethylase Activity

*E. coli* cells were harvested and lysed, and crude cell extracts were prepared as described in our previous work (Jahanian et al. 2024). Thebaine 6‐*O*‐demethylase (T6ODM) activity was measured in cell lysates using a thebaine consumption‐based assay in potassium phosphate buffer (K_2_HPO_4_) at pH 7.4 containing α‐ketoglutarate, sodium ascorbate, iron (II) sulfate heptahydrate, and thebaine. Reactions were incubated at room temperature quenched by thermal inactivation and analyzed by liquid chromatography with tandem mass spectrometry (LC‐MS/MS).

### 2.4 Codeinone Reductase and Isocitrate Dehydrogenase Activities

Codeinone reductase (COR) and isocitrate dehydrogenase (IDH) activities were determined by monitoring NADPH production at 340 nm, following previously reported methods (Jahanian et al. 2024). Enzyme activities were calculated using the Beer–Lambert law, with one unit (U) defined as the formation of 1 µmol NADPH per minute.

### 2.5 LC‐MS/MS Analysis

#### 2.5.1 Alkaloid Quantification

Alkaloid quantifications for the bioconversion of thebaine to codeine were analyzed by liquid chromatography, as described previously (Jahanian et al. 2024). Analyses were performed using a triple‐quadrupole mass spectrometer (LC‐MS/MS) model Shimadzu‐8050 triple‐quadrupole with Onyx Monolithic C18 column (100 × 4.6 mm^2^, Phenomenex Australia Pty Ltd.) was used for all samples and standards, with an injection volume of 1 μL. Analytes were eluted with an increasing linear gradient of 0%–20% (v/v) solvent B over 10 min (solvent A: 0.1% formic acid (FA) in water, solvent B: 0.1% formic acid (FA) in acetonitrile), at a flow rate from 1 to 1.5 mL·min^–1^ and at an oven temperature of 40°C. Analytes were detected using a UV detector at *λ* = 285 nm.

#### 2.5.2 Metabolomic Analysis

Targeted intracellular metabolite analysis was performed by LC‐MS/MS using methods adapted from (McDonald et al. 2017; Medina‐Torres et al. 2015). Analyses were conducted using a Dionex Ultimate 3000 HPLC system coupled to an AB Sciex 4000 QTRAP mass spectrometer. Chromatographic separation was performed on a Phenomenex Gemini‐NX C18 column (150 × 2 mm, 3 µm, 110 Å) equipped with a SecurityGuard Gemini‐NX C18 guard column at 55°C, using a 50 min gradient at a flow rate of 300 µL min^−1^. The mobile phases consisted of 7.5 mM aqueous tributylamine adjusted to pH 4.95 with acetic acid as solvent A and acetonitrile as solvent B. Samples were maintained at 4°C in the autosampler, and 10 µL was injected for analysis. Metabolites were detected in negative ionization mode using scheduled multiple reaction monitoring. Quantification was performed using calibration curves prepared from serial dilutions of analytical standards, with azidothymidine used as an internal standard. Standard mixtures and pooled quality‐control samples were injected throughout the analytical sequence to monitor instrument performance and data quality. Data was processed using *MultiQuant 2.1* software.

### 2.6 Formulation, Spray Drying, and Storage of *E. coli* Cells

Spray drying of the formulated cells was carried out using a Buchi‐B290 lab‐scale spray dryer (Buchi, Switzerland) with up to 1 L/h capacity, 2–25 µm particle size range, maximum inlet temperature of 220°C, and overall dimensions of 650 × 1100 × 700 mm. Harvested cells from fermentation were washed with cold phosphate‐buffered saline (PBS) solution and the cell pellet was re‐suspended in water to reach a final concentration of 10 g DCW.L^−1^. Maltodextrin at concentrations of 1% w/v and 2% w/v, and calcium chloride at a concentration of 0.2% w/v (total carrier concentrations: 1.2% and 2.2% w/v) were used as protective carriers. This formulation has been confirmed to provide high water solubility and microencapsulation efficiency (Zhang et al. 2018). A design of experiments was developed using inlet air temperatures ranging from 80°C to 140°C and carrier concentrations of 0%–2.2% to optimize drying yield and residual enzyme activity. This range was selected to evaluate the effects of drying severity and carrier loading, as higher inlet temperatures reduced the final moisture content, while carrier addition generally improved powder recovery compared with the no‐carrier condition. Operational conditions of the spray dryer, including feed rate, inlet temperature, atomization pressure, and outlet temperature, were adjusted based on the imposed inlet temperature. The spray‐dried powder was used for residual activity tests and storage stability studies. Total recovery yield was defined as the ratio between the obtained powder after spray drying and the total solid content in the bacterial suspension before spray drying. Dry‐weight basis moisture content (*M*_db_) was calculated based on the wet powder weight loss divided by dry powder weight of samples treated in an oven at 85°C for 24 h. Powders were stored for up to 3 months at two temperatures, 4°C and 24°C, in the dark. The stability of the spray‐dried product was investigated every 30 days by testing enzyme activity.

### 2.7 Thermal Inactivation and Kinetic Parameters Estimation

Aliquots (250 µL) of the resuspended dried cells and lysate formats in triplicate were transferred to 1.5 mL tubes and subjected to heat inactivation process at temperatures ranging from 40°C to 70°C for incubation periods sufficient to monitor the decline in residual enzyme activity. The samples were removed and immediately cooled to the assay temperature and the specified enzyme activity was measured as described above. The experimental data fitted to Equation 1.

(1) $$\ln\left(A\right)={\ln(A}_{0})-\mathrm{kt}\;$$

where *A/A*_0_ represents the residual relative enzyme activity after heat treatment time *t* (min), and *k* (min^−1^) is the inactivation constant at a particular temperature. The half‐life (*t*_1/2_) of inactivation was calculated as $t_{1/2}=\frac{\ln2\;}{k}$. Decimal reduction time (D‐value) is defined as the time through which the initial activity of the enzyme was reduced by 90% according to the equation: $D=\frac{\ln(10)}{k}$ (Thapliyal et al. 2018).

### 2.8 Intracellular Metabolite Extraction

The intracellular metabolites were determined for *E. coli* cells at different growth times during batch cultivations in triplicate (Au et al. 1989; Luna‐Flores et al. 2018; Ser et al. 2015). Briefly, 1 mL of the cells were normalized at 0.64 g.L^−1^ DCW was centrifuged at 20,000*×g*, and 4°C for 2 min. The supernatant was discarded and the cell pellets were placed on ice (4°C). Subsequently, the pellet was resuspended in 1 mL of extraction solvent (50% acetonitrile (ACN)), precooled in a –80°C freezer, and the solution was vortexed for 10 s with three cycles with 2 min of cooling on ice in between these cycles. The samples were then centrifuged at 20,000*×g* and 4°C for 3 min. The supernatant (900 µL) was transferred to another tube and frozen at –80°C before freeze‐drying into powder. Finally, the powder was resuspended in 100 µL of water containing 2% ACN and 5 µM azidothymidine as an internal standard to be analyzed by LC‐MS/MS.

### 2.9 Whole‐Cell Recycling Bioconversion

*E. coli* BL21 cells producing T6ODM‐NISO or COR enzymes were harvested from batch cultivation in defined minimal medium at 23°C for 24 h, as described previously (Jahanian et al. 2024). The cells were washed twice with cold PBS buffer and spray‐dried under the optimized conditions. Spray‐dried powder was then rehydrated in reaction buffer to obtain a final cell loading normalized to a specific activity of 15 U.L^−1^ for each T6ODM and COR biocatalyst. Each bioconversion reaction was freshly prepared in 100 mM phosphate buffer (pH 6.0) containing 0.5 mM thebaine, 0.5% (w/v) glucose, 5 mM sodium ascorbate, and 10 µM each of FeSO_4_, MnCl_2_, and MgSO_4_, in a total volume of 10 mL. In the first step, spray‐dried cells producing T6ODM‐NISO were resuspended in the reaction solution to convert thebaine to codeinone/neopinone. The resulting supernatant was then transferred to rehydrated spray‐dried cells producing COR to complete the conversion of codeinone/neopinone to codeine and neopine, as shown in Figure 1a. All reactions were incubated for 30 min at 24°C and 220 rpm. At the end of each cycle, the biocatalyst cells were recovered by centrifugation at 16,000×*g* for 10 min at 4°C and directly resuspended in fresh reaction solution for the next cycle. No additional pretreatment, washing, or reactivation was applied between cycles. Fractions of the cells and supernatant were collected at the end of each cycle for residual enzyme activity measurement, intracellular metabolite analysis, and bioconversion yield determination.

## 3 Results

### 3.1 Optimization of Spray Drying Whole *E. coli* Cells

The operational conditions of the spray dryer were adjusted for the spray dryer based on four different inlet air temperatures (80°C, 100°C, 120°C, and 140°C) to achieve a high‐quality dried cell powder with optimized enzyme activity retention. Operating at inlet temperature below 80°C was unsatisfactory, as the resulting high residual moisture content could lead to poor storage stability (data not shown). Other studies have shown that a residual moisture content of 2%–3% can be optimal for the long‐term storage of biocatalyst powders (Behboudi‐Jobbehdar et al. 2013; Chang et al. 2005). The results presented here (Table 1) showed that spray‐dried powders generated at an inlet temperature of 80°C had a maximum residual moisture content of 4.3% w/w under the given conditions.

**Table 1:** Operational conditions and yields of spray‐dried *E. coli* (BL21) cells producing T6ODM/NISO and COR with different inlet air temperatures.

Inlet air temperature (°C)	Feed flow rate (mL.min^–1^)	Outlet air temperature (°C)	Atomization pressure (mm Hg)	Actual moisture content (%) (dry weight based)	Total recovery yield (%)		
					No carrier	1.2% carrier	2.2% carrier
80	2.9	42 ± 1	3.5	4.3 ± 0.5	70.0 ± 1.9	82.0 ± 1.5	71.0 ± 0.8
100	5.5	47 ± 1	3.5	3.9 ± 0.3	68.6 ± 0.9	77.1 ± 1.3	85.0 ± 0.9
120	7.7	52 ± 1	3.0	3.3 ± 0.2	66.0 ± 2.1	72.3 ± 1.4	82.1 ± 0.8
140	9.6	57 ± 1	3.0	2.7 ± 0.1	61.3 ± 1.4	68.6 ± 0.8	78.6 ± 0.8

*Note:* Values represent the average of three replicates of washed *E. coli* cells resuspended in water with or without protective carrier agents under the given operational conditions. Standard deviations are shown.

Table 1 shows that inclusion of the carrier agent, a mixture of maltodextrin and calcium chloride, was important as the total solid recovery yield was below 70% in the absence of a carrier at all temperatures. However, a maximum yield of 85% was achieved when 2.2% of the protective carrier was used at an outlet air temperature of 47°C. Higher concentrations of protective carrier were not further considered, since they caused high viscosity in the rehydrated biocatalysts, which reduced the biocatalytic reaction yield and created additional challenges for downstream purification.

**Figure 2:**
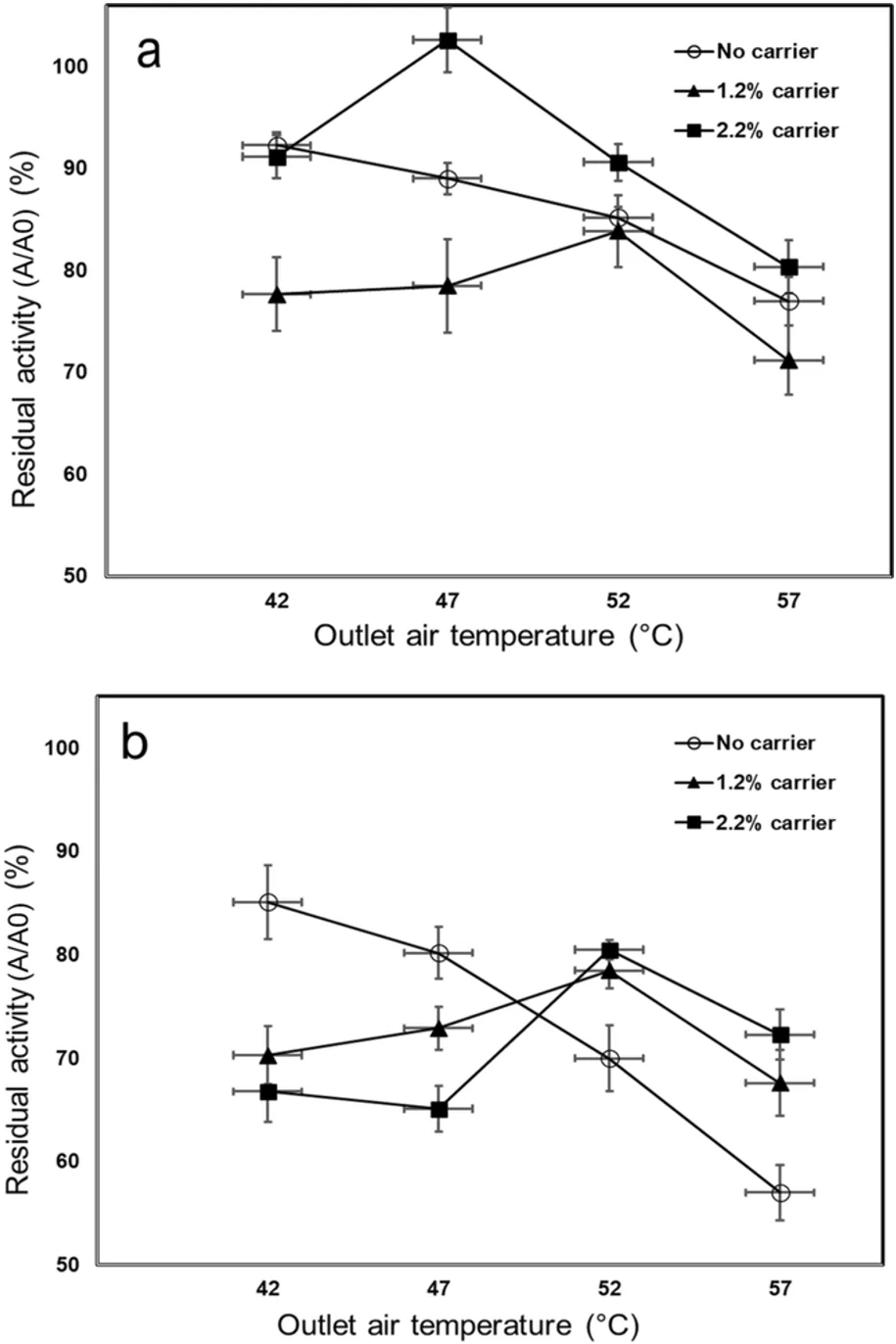
Residual activity of (a) T6ODM and (b) COR enzymes under various outlet air temperatures and carrier (maltodextrin‐CaCl_2_) concentration (% w/v).

Figure 2 shows the influence of the spray drying temperature on the residual activity of T6ODM and COR for each operational condition described in Table 1. In the absence of a carrier, residual activity decreased progressively with increasing outlet temperature, suggesting that thermal stress was the dominant factor controlling enzyme activity loss. In contrast, carrier‐containing formulations showed an optimum temperature, indicating a balance between sufficient drying, carrier‐mediated protection, and thermal damage. Below the optimum, incomplete drying or insufficient matrix formation may limit protection, while above the optimum, thermal inactivation becomes dominant. No residual activity loss was evident for T6ODM when spray drying occurred at a 47°C outlet air temperature with 2.2% of protectant carrier. However, lower T6ODM residual activity observed with 1.2% carrier compared with the no‐carrier condition suggests that the protective effect of the maltodextrin–CaCl_2_ formulation was concentration‐dependent rather than linear. At 1.2%, the carrier level may have been insufficient to form an effective protective matrix around the cells during drying and rehydration, whereas 2.2% carrier provided improved protection, giving the highest T6ODM residual activity at an outlet temperature of 47°C (Figure 2a). For the COR enzyme, the maximum residual activity of 80.5% occurred at 52°C with the same carrier concentration (2.2%).

### 3.2 Thermal Inactivation Analysis of Enzymes (Whole Cell vs. Cell Lysate)

**Figure 3:**
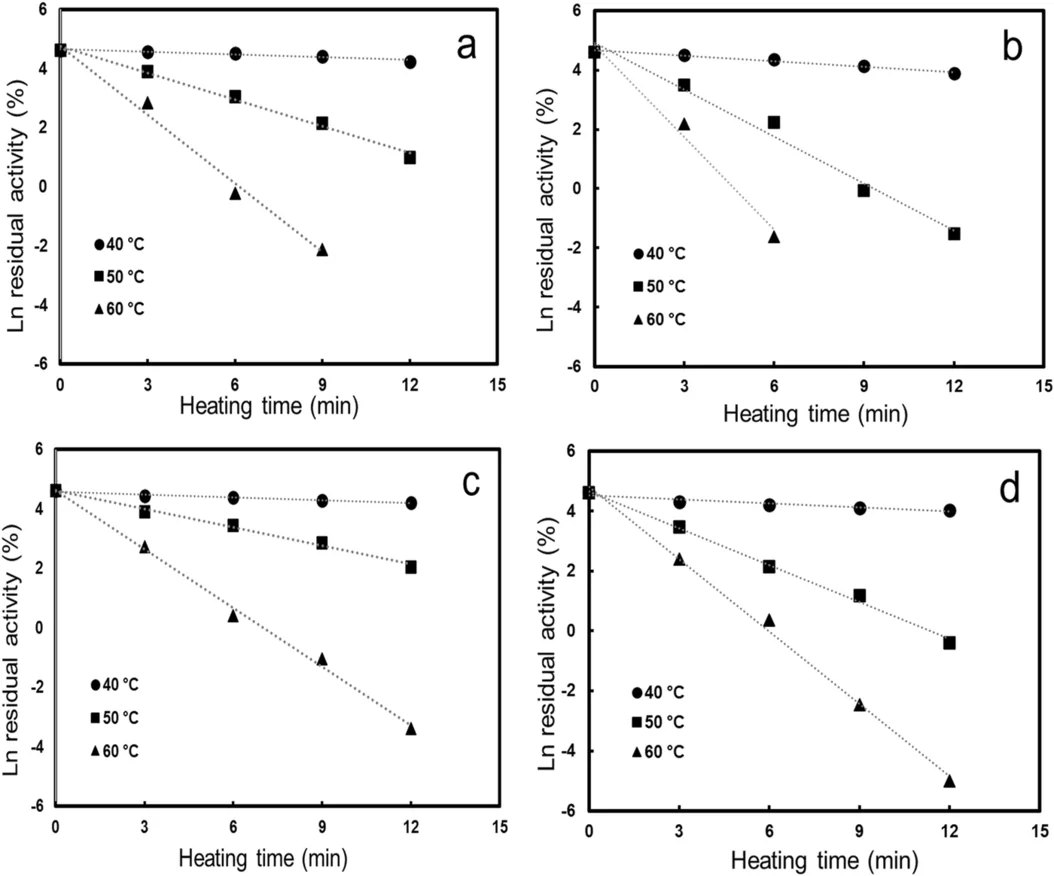
Thermal inactivation test represented as the natural logarithm of residual activity of thebaine‐6‐*O*‐demethylase (T6ODM) in formats of (a) whole cells, (b) lysed cells; and codeinone reductase (COR) in the format of (c) whole cells, and (d) lysed cells for the incubation temperature at 40°C, 50°C, and 60°C.

The semi‐log graphs plotted from the residual activity vs. heating time were linear in the range of 40°C–60°C for T6ODM and COR enzymes in the two formats of whole‐cells and cell lysates (Figure 3). Preliminary experiments showed that both T6ODM and COR had no detectable residual activity after 12 min at 50°C, whereas enzyme inactivation at 70°C occurred within less than 3 min (data not shown). Inactivation constants (*k*), based on exponential activity loss, were determined from the slope of inactivation curves, indicating that the thermal inactivation of both enzymes fitted a first‐order monophasic kinetic model (*R* ^2^ = 0.90–0.99) (Figure 3). The thermal stability of the enzymes can be represented by the terms of half‐life time (*t*_1/2_) and decimal reduction time (D‐value) (Thapliyal et al. 2018). The calculated *t*_1/2_ and D‐values in Table 2 indicate that COR generally had a slightly higher thermal resistance compared to T6ODM at all tested temperatures, which was more pronounced at higher temperatures of 50°C and 60°C. Proteins inside the intact cells were more resistant to heat denaturation. For example, Table 2 indicates that at 40°C the inactivation half‐life for T6ODM and COR were 50% and 31% higher, respectively, when presented in whole cell than in lysate. Similarly, the D‐value of T6ODM and COR increased twofold and 1.5‐fold inside the intact cells compared with lysates. The *E. coli* cell membrane has been reported to be stable at temperatures up to 49°C and enzymes are expected to be more protected within the cellular environment than when exposed in solution (Kwon et al. 2008), consistent with these observations.

**Table 2:** Kinetic parameters of thermal inactivation, including the inactivation constant (*k*) with associated *R* ^2^, half‐life (*t*_1__/__2_), and decimal reduction time (*D*‐value), for thebaine 6‐*O*‐demethylase (T6ODM) and codeinone reductase (COR) in whole‐cell and lysate formats.

Enzyme format	Temp (°C)	T6ODM				COR			
		*k* (min^−1^)	*R* ^2^	*t*_1/2_ (min)	*D* (min)	*k* (min^−1^)	*R* ^2^	*t*_1/2_	*D* (min)
Whole cell	40	0.03 ± 0.00	0.90	23.41	77.80	0.03 ± 0.00	0.97	22.14	73.76
	50	0.30 ± 0.02	0.99	2.31	7.70	0.20 ± 0.00	0.99	3.38	11.20
	60	0.77 ± 0.01	0.98	0.89	2.97	0.65 ± 0.01	0.99	1.05	3.50
Cell lysate	40	0.06 ± 0.00	0.97	11.55	38.38	0.040 ± 0.00	0.91	15.26	50.71
	50	0.53 ± 0.02	0.98	1.31	4.37	0.41 ± 0.02	0.99	1.69	5.63
	60	1.03 ± 0.04	0.98	0.67	2.22	0.80 ± 0.020	0.99	0.86	2.87

### 3.3 Storage Stability of the Enzymes

**Figure 4:**
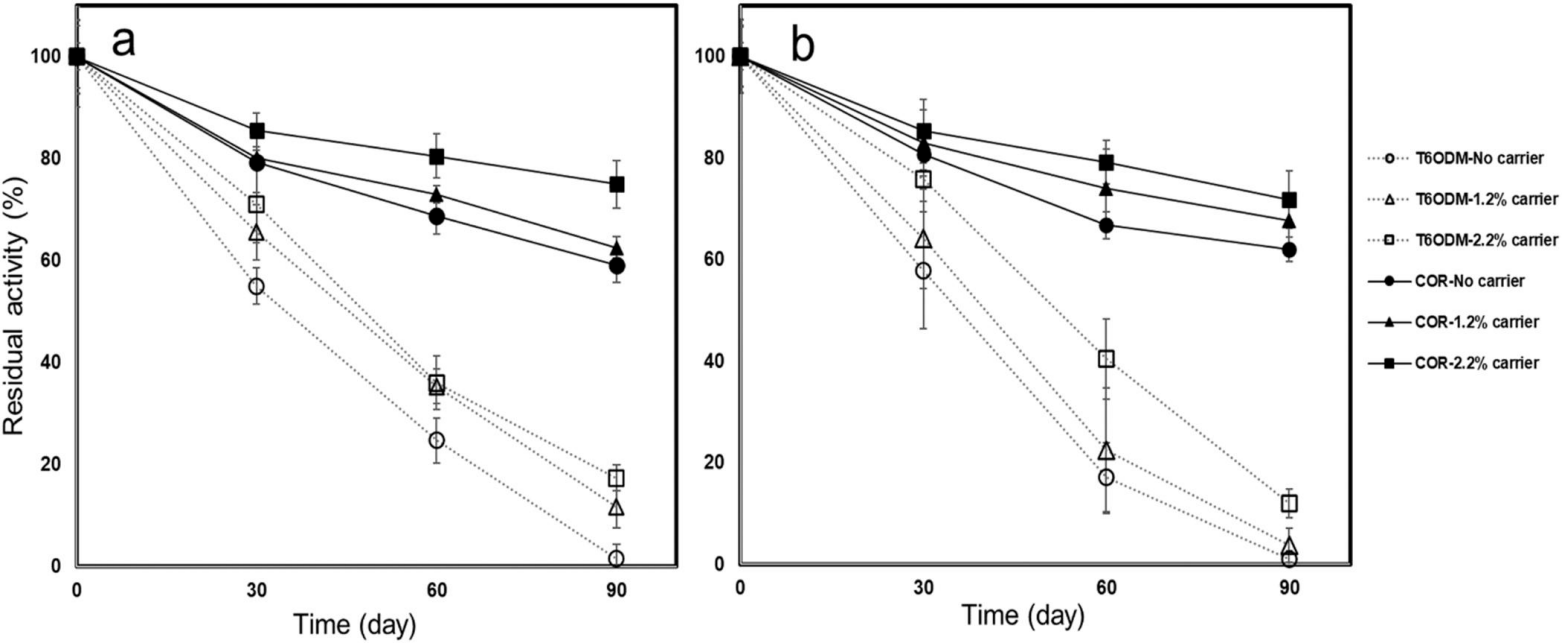
Residual activity of T6ODM and COR in spray‐dried cells during storage at (a) 4°C, and (b) 24°C. The corresponding absolute activities at 100% were 14.33 U.g^−1^ DCW for T6ODM and 42.61 U.g^−1^ DCW for COR. Error bars represent the average of three independent trials.

In the storage stability studies presented here for spray‐dried cells, there was a general decline in enzyme activity over time, while storage temperature, either refrigerated (4°C) or room temperature (24°C), did not strongly affect the rate of decline or the residual activity of the formulated enzymes. T6ODM showed lower storage stability over time, consistent with its inherent lower stability compared with COR, and its residual activity reached zero after 90 days in samples without a carrier (Figure 4). The addition of maltodextrin‐CaCl_2_ as the carrier had a positive effect on the long‐term survivability of both enzymes in comparison to storage without a carrier. For example, after 90 days storage the application of 2.2% carrier in the formulation of the cells improved the T6ODM and COR stability by around 20% compared to storage without a carrier. In contrast to T6ODM, COR showed more resistance to storage, losing 40% of its initial activity after 90 days of storage without a protectant carrier, while in the presence of 2.2% protectant carrier, the residual enzyme activity was 75% and 72% at storage temperatures of 4°C and 24°C, respectively. Overall, the difference between the refrigerated storage and room temperature was not significant for the stability of either T6ODM or COR in dried cells (Figure 4).

### 3.4 Intracellular Metabolite Levels in Growing and Harvested Resting Cells

**Figure 5:**
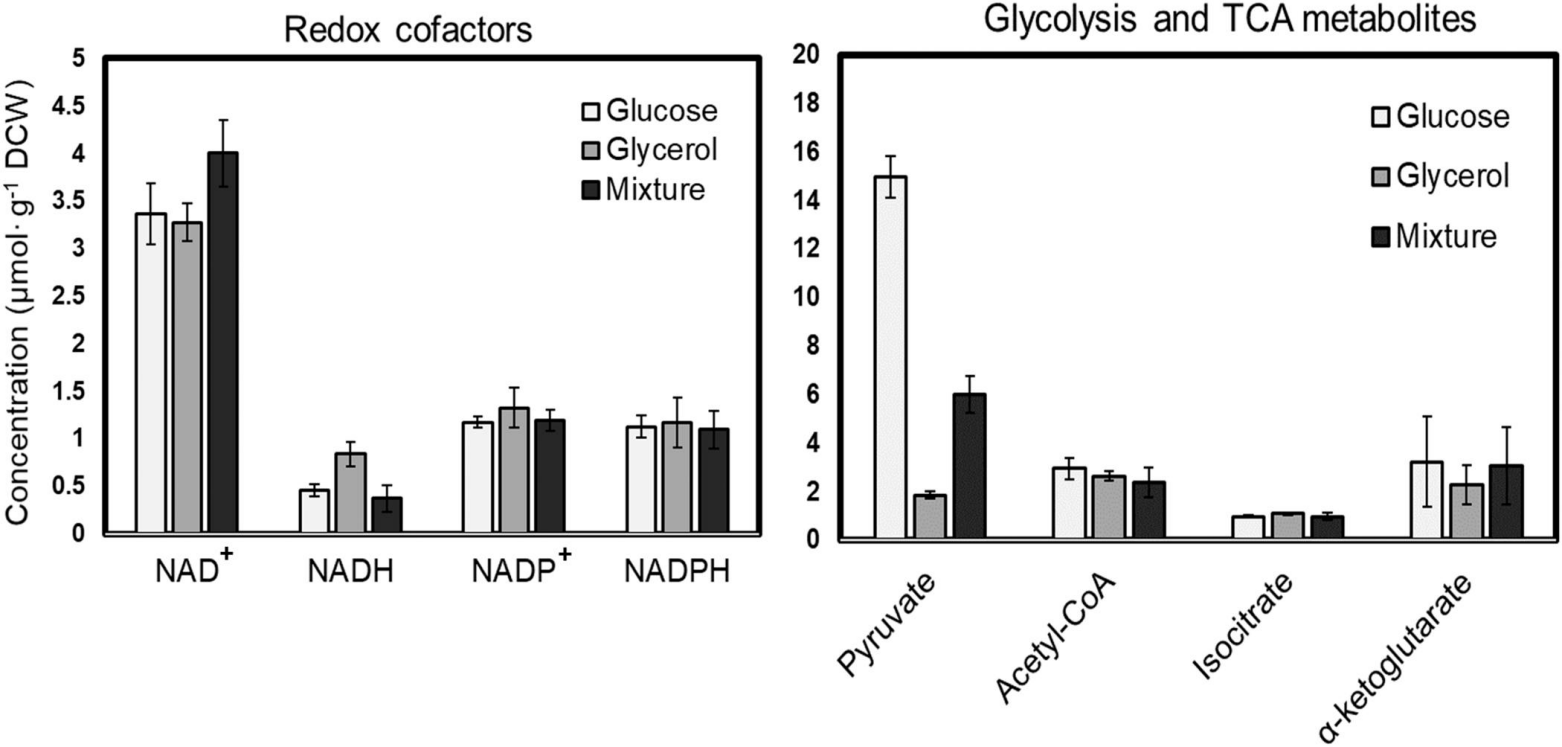
Abundance of intracellular metabolites in *E. coli* BL21(DE3) showing the effect of carbon source (glucose, glycerol, and a 50/50 mixture of both), measured in stationary phase after 24 h. Data represents the mean of three independent batch growth trials, and error bars represent the standard deviation.

Intracellular metabolite titers during microbial growth can vary significantly depending on the growth phase, carbon source, and fermentation conditions. In this study the impact of carbon source and growth phase was studied on nicotinamide redox cofactors and glycolysis and tricarboxylic acid cycle (TCA) metabolites levels. Figure 5 shows that the use of glucose or glycerol as the carbon source or an equal mixture of both did not lead to a significant difference in the titer of nicotinamide redox cofactors and isocitrate at the end of fermentation (*t* = 24 h). NADPH levels in all given carbon source regimes remained around 1 µmol.g^−1^ DCW. The levels of α‐ketoglutarate, one of the key intermediates in the TCA cycle and the co‐substrate for T6ODM (Lozano Terol et al. 2019), did not vary considerably when using either of these carbon sources.

**Figure 6:**
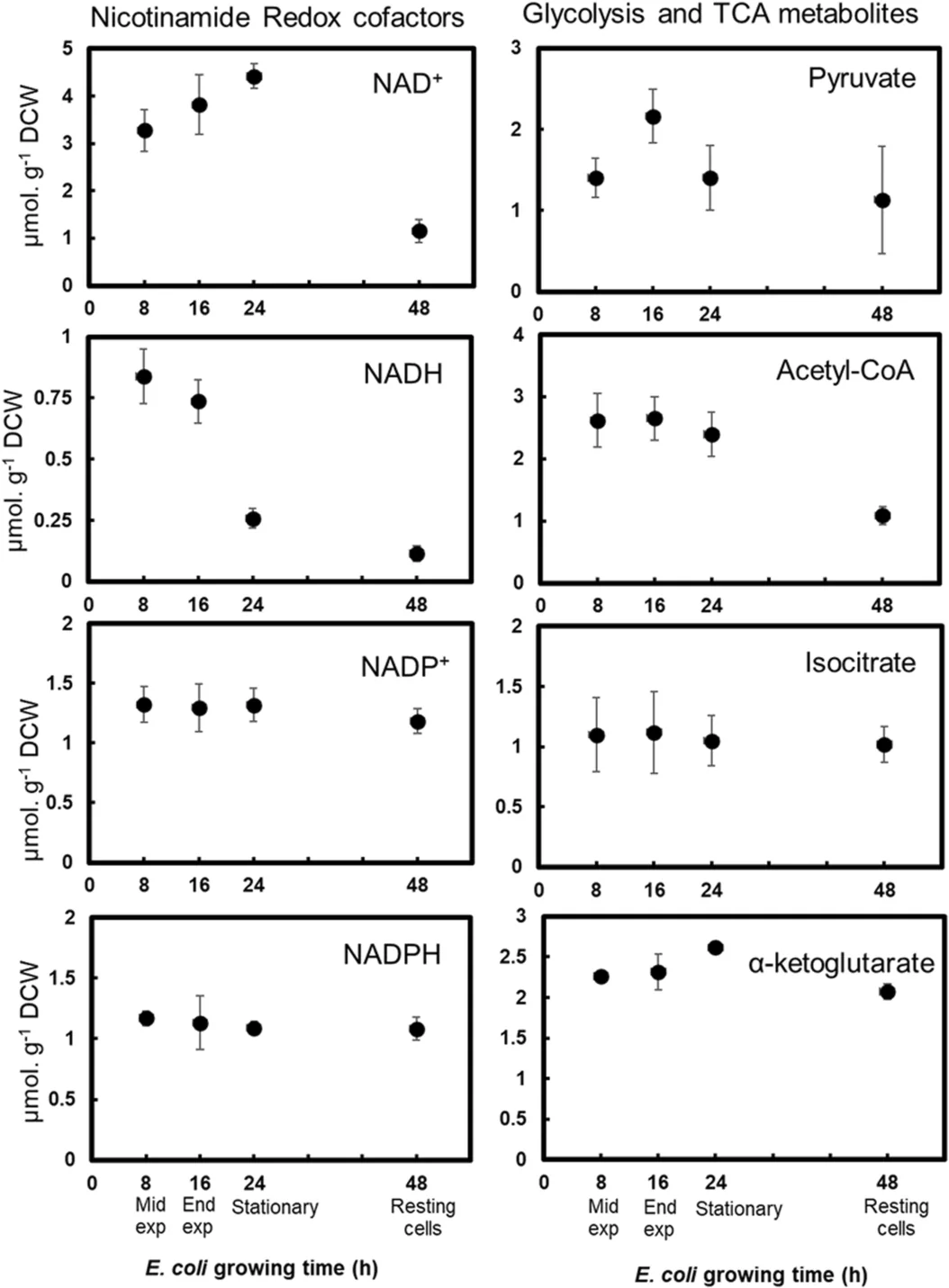
Abundance of intracellular metabolites in *E. coli* BL21(DE3) at different growth phases when cultured on glycerol. Data represents the mean of three independent batch growth trials, and error bars represent the standard deviation.

Figure 6 shows that the concentration of NADP^+^/NADPH, isocitrate, and α‐ketoglutarate was minimally affected by different bacterial growth phases and did not decrease in resting cells after 48 h of fermentation. In contrast, NAD^+^/NADH cofactors showed a significant decrease in resting cells compared with the growing phase. During the growing phase, there was a notable shift in cofactor balance, with an increase in the oxidized form (NAD^+^) and a corresponding decrease in the reduced form (NADH). This dynamic change in the redox state may influence various metabolic pathways, especially those dependent on NAD^+^/NADH ratios, affecting the efficiency of dehydrogenase enzymes and other redox reactions critical to cellular metabolism (Figure 6). Pyruvate and acetyl‐CoA levels also varied across growth phases in response to metabolic demand. During exponential growth, high glycolytic flux supports pyruvate turnover and conversion to acetyl‐CoA for biomass formation and energy metabolism. At stationary phase, reduced anabolic demand was associated with lower pyruvate levels compared with the preceding growth phase, while acetyl‐CoA levels remained relatively stable. In resting cells, overall metabolic activity was reduced, and the relatively large variation observed for pyruvate at 48 h may reflect biological differences in residual metabolic activity and the sensitivity of this dynamic central‐carbon intermediate to sampling and extraction. In resting cells, overall metabolic activity is reduced, and pyruvate and acetyl‐CoA primarily support cellular maintenance rather than growth; therefore, the relatively large variation observed for pyruvate at 48 h may reflect biological differences in residual metabolic activity and the sensitivity of this dynamic central‐carbon intermediate to sampling and extraction.

### 3.5 Enzyme Stabilities and Cofactor Levels in Recycled Whole Cell Biocatalysts in Sequential Batch Reactions

Spray‐dried whole cells were rehydrated in phosphate buffer and supplemented with glucose, which was not used as a carbon source for biomass growth but rather as a substrate for intracellular enzymes in central carbon metabolism to facilitate the generation of the required NADPH and α‐ketoglutarate for the bioconversion. Thebaine was added to T6ODM‐producing cells as the substrate for the first step of the bioreaction, resulting in the production of codeinone and neopinone. These intermediates were then introduced into the second step of the bioreaction, involving COR‐producing cells, to convert them into codeine and neopine.

**Figure 7:**
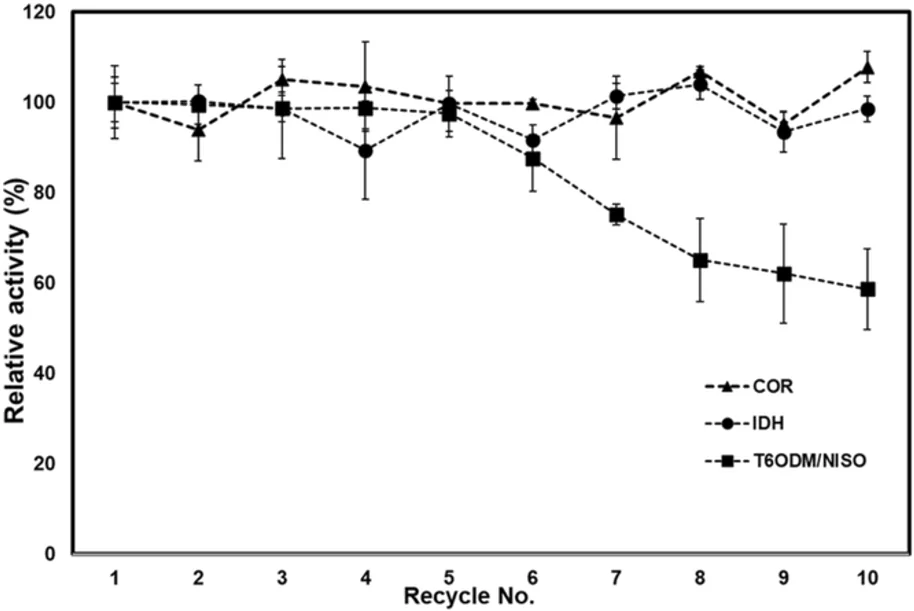
Relative activity of enzymes in whole cell biocatalysts recycling over up to 10 biotransformation cycles. Error bars represent the standard deviation of three independent experiments.

At the end of each 15‐min reaction cycle, the cell pellets were collected after centrifugation and subjected to fresh substrate and buffer. As Figure 7 shows, the residual activity of COR and isocitrate dehydrogenase (IDH) was maintained in the *E. coli* cells at the end of 10 recycles under the given reaction conditions (i.e., pH 6 and 24°C), indicating these enzymes are highly stable in repetitive bioconversions. In comparison, T6ODM maintained activity after five recycles but lost around 40% of its initial activity at the end of 10 recycles, which is not unexpected, due to the lower stability of T6ODM than COR. The average IDH activity between recycles in T6ODM and COR harboring cells was shown to be 23 U.g^−1^ DCW and 18 U.g^−1^ DCW, respectively.

**Figure 8:**
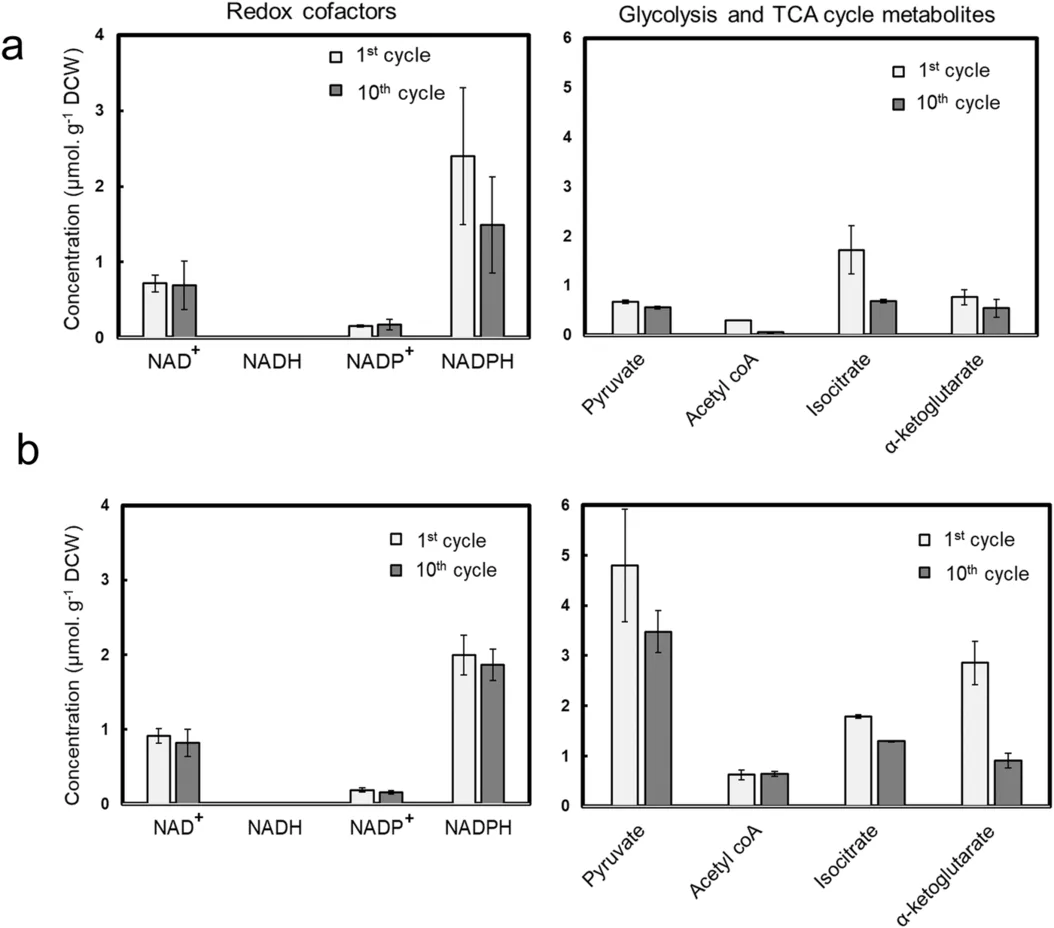
Intracellular metabolite concentrations at the end of the reaction from the first and last whole‐cell recycling of *E. coli* BL21 resting cells of (a) T6ODM producing cells, (b) COR producing cells. Error bars represent the standard deviation of three independent biological reactions.

Metabolomic analysis of nicotinamide redox cofactors throughout the recycling cycles showed that NADPH remained relatively high in both T6ODM‐ and COR‐producing cells, whereas NADH remained low (Figure 8). A larger variation in NADPH was observed in T6ODM‐producing cells, while COR‐producing cells showed more consistent NADPH levels. The higher variability in T6ODM‐producing cells may reflect biological differences in NADPH regeneration and turnover during the oxygen‐ and α‐ketoglutarate‐dependent reaction. This balance can be explained by the fact that the function of redox cofactors is different. NADP^+^/NADPH is used for modulating anabolic redox reactions, whereas NAD^+^/NADH is used for oxidation reactions (Miller et al. 2018). Therefore, as Figure 8 shows, it is expected that the cells restore NADP^+^/NADPH in a reduced form and NAD^+^/NADH in an oxidized form. Despite some differences in intracellular metabolite levels between T6ODM‐producing cells and COR‐producing cells, the concentration of α‐ketoglutarate (co‐substrate for T6ODM), remained stable after 10 cycles of biotransformation.

**Figure 9:**
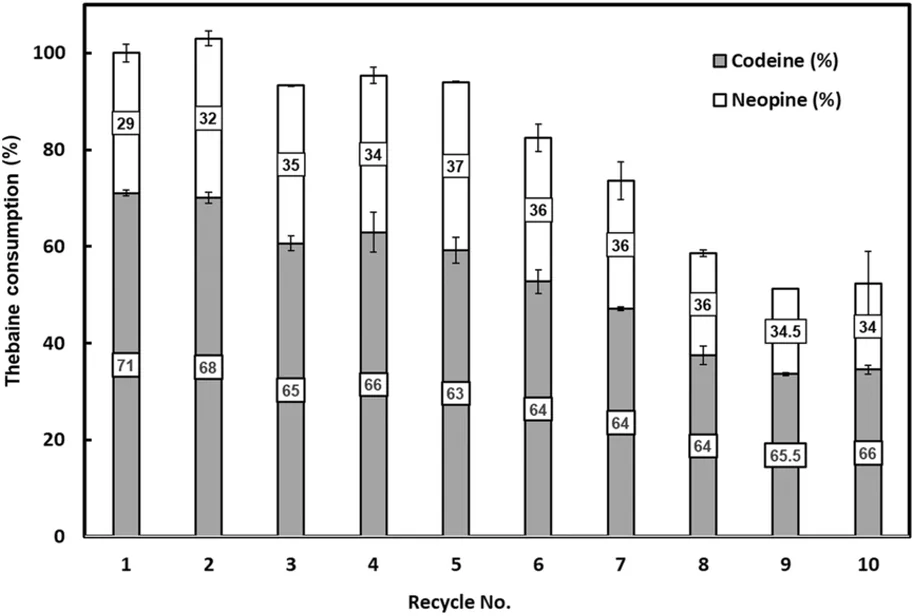
Substrate (thebaine) consumption and codeine/neopine final ratios in two‐step reaction cycles (15 min each) catalyzed by whole *E. coli* cells producing thebaine 6‐*O*‐demethylase (T6ODM) and neopinone isomerase (NISO), followed by cells producing codeinone reductase (COR).

The two‐step recycling use of *E. coli* was conducted to convert thebaine to codeinone/neopinone using T6ODM producing cells, then to codeine/neopine using COR producing cells using equally normalized enzymatic activities. The final codeine to neopine ratio in all recycles fluctuated between 63:37 and 71:29, (Figure 9). These ratios generally reflected the equilibrium position between codeinone and neopinone, approximately 60:40. This observation is likely due to the design of the experiment, where equilibrium was reached in the first step before COR was added to the codeinone/neopinone mixture. The bioconversion yield in the given reaction time (15 min + 15 min) decreased gradually to 54% after 10 recycles (Figure 9), likely due to the reduced catalytic activity of T6ODM, as the reaction intermediates codeinone/neopinone were not identified after 15 min of the COR catalyzed reaction, for all recycles. Complete thebaine conversion could be achieved by extending reaction times (i.e., to 30 min) or by using higher loadings of T6ODM producing cells.

## 4 Discussion

The main objective of this study was to develop a durable biocatalyst formulation for scalable codeine biomanufacturing from thebaine. In our previous study we optimized growth media, induction method, and fermentation conditions to maximize the activity of T6ODM and COR enzymes, which led to an 80% yield of codeine in a single biotransformation step using freshly prepared whole‐cell biocatalysts (Jahanian et al. 2024). Here, we show that spray‐dried formulated *E. coli* cells producing T6ODM (co‐produced with NISO) and cells producing COR were able to preserve residual enzyme activity and display thermostability in whole‐cell formats. The protective effect of the maltodextrin–CaCl_2_ carrier on bacterial cells is likely associated with formation of a carbohydrate‐rich protective matrix around the bacterial cells, which reduces dehydration, thermal, and oxidative stresses during spray drying and helps preserve membrane integrity and intracellular enzyme structure (Perdana et al. 2014; Semyonov et al. 2010). The storage stability of enzyme biocatalysts is important for commercial enzyme applications (El‐Sherbiny and El‐Chaghaby 2011). The stability of enzymes during long‐term storage can be influenced by damaging environmental conditions, such as moisture and high temperature (Iyer and Ananthanarayan 2008). A glass transition temperature (*T_g_*) is a specific temperature for a compound in which all amorphous materials evolve from the glassy to soft elastic status. This glass like state of a substance relies on the temperature and moisture content, which means applying specific conditions is essential to store spray‐dried enzymes. Temperatures over the *T_g_* should be avoided, as the protein may unfold. Suitable storage temperatures should ideally be 50°C below the *T_g_* (Alhalaweh et al. 2015; Shrestha et al. 2007).

T6ODM is a dioxygenase enzyme that catalyzes the incorporation of both atoms of O_2_ into substrates. Dioxygenases can utilize either a non‐heme form of iron or flavin cofactor to activate molecular O_2_ or organic molecules in a controlled manner (Singh et al. 2021; Wang et al. 2017). T6ODM activity requires α‐ketoglutarate, which is converted to succinate during the reaction (Farrow and Facchini 2014), while the regeneration of α‐ketoglutarate from succinate with a cost‐effective method has not yet been reported. Oxygen is essential for dioxygenase activity, and elevated concentrations can increase oxidative stress on the enzymes, potentially compromising their performance (Busch et al. 2020). The stability and storage results show that T6ODM is more sensitive to thermal inactivation and long‐term storage compared with COR. It is possible that the oxygen‐dependent nature of T6ODM may be connected to its low storage stability, even when using a formulated format with a 2.2% protectant carrier. Runguphan et al. redesigned and translated codeine O‐demethylase (CODM), another enzyme from the dioxygenase family involved in morphinan alkaloid conversions, in *E. coli* and also demonstrated this enzyme was not stable for extended storage times (Runguphan et al. 2012). The molecular docking and molecular dynamics simulations of the T6ODM‐substrate complexes suggest that a 1:2 stoichiometric ratio for enzyme: substrate, where the first substrate molecule binds to the metal center and other binds at the distal site, forms a stable complex. In contrast, in a 1:1 T6ODM‐substrate stoichiometric complex model, the substrate shows reduced affinity to remain proximal to the metal cofactor, indicating lower stability in this configuration (Kachhap et al. 2020).

Non‐growing cells play a significant role in industrial biotechnology since they contain active enzyme to convert substrate into desired products and can also remain metabolically active and able to regenerate cofactors (Lempp et al. 2020). In this approach, cell growth and enzyme production are separated from catalysis and product synthesis, allowing finer control over biocatalyst loadings compared with metabolically engineered growing cells (Burg et al. 2016). Here, we show that the required intracellular metabolites were present in the harvested cells, but at much lower concentrations than would be required to support the subsequent biocatalytic reactions without active cellular metabolism. Accordingly, α‐ketoglutarate and NADPH regeneration by the resting cells during biotransformation was required to complete the reactions. The work of Walton and Stewart showed that in non‐growing *E. coli* cells, the IDH enzyme through the citric acid cycle is the major producer of NADPH, accounting for 83% of the total NADPH production (Walton and Stewart 2004). According to Takahashi et al, the activity of produced NADP^+^ linked IDH in recombinant *E. coli* is 40 times higher than cell‐free extract enzyme, and its activity is important in controlling the metabolic flux through the generation of α‐ketoglutarate and NADPH (Spaans et al. 2015; Takahashi‐Iñiguez et al. 2014). The similar level of IDH observed here therefore indicates that NADPH production is similar in the two cell lines and that the relative activity measured reflects enzyme stability rather than co‐factor availability.

Successful regeneration by the rehydrated cells during repeated reaction cycles was supported by supplying glucose to central carbon metabolism. When glucose is supplied, NADPH can be generated through glucose‐6‐phosphate dehydrogenase (G6PD) and 6‐phosphogluconate dehydrogenase (6PGD) in the pentose phosphate pathway, as well as through isocitrate dehydrogenase (IDH) in the citric acid cycle. In contrast, when glycerol is used as the carbon source, NADP^+^ reduction is mainly limited to IDH, which may result in lower NADPH turnover. IDH also contributes to α‐ketoglutarate biosynthesis in the citric acid cycle. Therefore, the maintenance of α‐ketoglutarate and NADPH during cell recycling suggests that the rehydrated resting cells retained sufficient metabolic activity to support co‐substrate and cofactor regeneration. This also indicates that the observed loss of T6ODM activity over repeated cycles was more likely related to enzyme instability rather than cofactor or co‐substrate limitation.

In summary, the biotransformation of thebaine to codeine using formulated spray‐dried cells was shown to be a promising scalable process with potential for industrial application. Whole cell biotransformation eliminates the need for costly supplementation or external regeneration of co‐substrate and cofactors, as they were effectively regenerated within the cells through carbon metabolism by the addition of glucose. The method developed here facilitates long‐term storage, transportation, and reuse of the biocatalyst – reducing fermentation cost and volumes by increasing the amount of product for each unit of biocatalyst. The findings established here can be used to develop a techno‐economic model to estimate the viability of an industrial codeine biomanufacturing process.

## Ethics Statement

The authors have nothing to report.

## Conflicts of Interest

The authors declare no conflicts of interest.

## Data Availability

Data sharing is not applicable to this article, as no new data were created or analyzed in this study.
